# MULTISECTOR PLANS FOR AGING: OPPORTUNITIES FOR AGING RESEARCH TO INFORM STATE POLICY

**DOI:** 10.1093/geroni/igae098.2205

**Published:** 2024-12-31

**Authors:** Valerie Flores, Carrie Graham

**Affiliations:** Center for Health Care Strategies.; Center for Health Care Strategies, Berkeley, California, United States

## Abstract

State Multisector Plans for Aging (MPA) are state-led, cross-department, stakeholder- and evidence-informed plans through which states set goals and priorities to transform their state and improve care, services and support for their diverse aging populations. Often these plans are led by the Governors’ offices, Departments of Aging or Medicaid agencies, but also engage across departments of public health, social services, emergency preparedness, housing, transportation, labor, and more. They are also informed by meaningful stakeholder engagement, including advocates, consumers, aging services providers and employers in the state. By far one of the most important MPA stakeholders are universities and researchers who can provide cutting edge data to inform MPAs. Currently, almost half of all U.S. states are engaged in either the development process or implementing an MPA. Evidence and data are important at every stage, from building the case for the necessity of an MPA, to the identification of unmet needs in the state, to development of strategies, and ultimately defining benchmarks and measuring progress. Many states have partnered with gerontological researchers to inform their plans. This offers the mutual benefit of informing the state, as well as providing an opportunity for researchers to learn how to better formulate their research questions to help the state build actionable and timely policy. This session will include an overview of how researchers have contributed to MPAs in many states and how the relationships forged through that process has opened more opportunities for continued collaboration and the translation of research into policy.

